# Impact of Cognitive Functioning and Age on Patient-Reported Outcomes in Parkinson’s Disease

**DOI:** 10.1093/geroni/igaa057.510

**Published:** 2020-12-16

**Authors:** Ann Gruber-Baldini, Haesung Kim, Jay Unick, Lisa Shulman

**Affiliations:** 1 University of Maryland School of Medicine, Baltimore, Maryland, United States; 2 University of Maryland School of Social Work, Baltimore, Maryland, United States

## Abstract

Cognitive impairment is prevalent in Parkinson Disease (PD) and increasing age is a PD risk factor. Age and cognition may impact patient-reported outcome measures (PROMs) level, reliability, or validity of responses. This study investigated the relative impact of cognitive function and age on PROMs in PD. Cross-sectional data (n=676) included assessments of age, cognition (Montreal Cognitive Assessment; MoCA) and PROMIS-29 Profile (physical functioning, anxiety, depression, fatigue, sleep disturbance, social functioning, pain). Analyses examined differences by age and MoCA in: 1)Level—correlations, multivariable regressions controlling for disease severity (UPDRSmotor, PD duration), comorbidity (CIRS-G), demographics; 2)Reliability--Cronbach’s alpha, and 3)Validity--correlations of PROMIS physical function with physician assessments. Sample was age M=68.0(SD=9.1); range=36-93 years, 64% male, 87% white, 37% college educated, PD duration M=8.2(SD=6.1) years, and MoCA M=24.3(SD=4.9; range 2-30). Greater cognitive impairment was consistently associated with greater physical/mental impairment (r=.14-.45; p<.05), except for sleep disturbance (r=-.07, p=.08) Multivariable regressions found cognition remained a significant predictor of physical functioning, anxiety, and depression older age predicted anxiety and social functioning. Comorbidity was the greatest predictor across all the PROMs (r=.22-.45). Reliability for PROMIS measures was excellent (alpha>.8) across cognitive and age groups, except for Fatigue at MoCA.36) across cognition and age groups. Cognitive impairment in PD is associated with lower physical function and mental health levels. Reliability and validity of most PROMs in PD are neither impacted by cognition nor age.

